# The Poor Man's Home.—V

**Published:** 1897-09-18

**Authors:** 


### MODERN SOCIOLOGY.

### THE POOR MAN'S HOME.—V.

### HEALTH LAWS IN MANY LANDS.

SANITARY laws vary greatly in different countries, according to the importance assigned to the subject by the authorities in each. Everywhere they are comparatively recent. The earliest were more or less tentative, and were often found faulty and incomplete in working. Hence came alterations and amendments of various kinds, till sanitary law became a subject too complicated for the plain man to deal with. In England there were till recently fourteen Acts dealing with one subject—the dwellings of the poorer classes; but these have been repealed, and their provisions, extended or modified as experience showed to be wise, have been consolidated in the Housing of the Working Classes Act, 1890.

This Act is divided into three parts. The first deals with unhealthy areas of considerable size and including a large number of houses; the second deals with the case of individual houses or small blocks of dwellings; the third gives authorities the right to erect lodging-houses for the working class, and to acquire or appropriate land for that purpose. We shall try to maintain these divisions in discussing the subject.

In Part I., "the leading idea is," says Mr. Wynter Blyth,* "to give sufficient power to the local authority to clear some well-defined unhealthy area in the city, and having removed the unwholesome dwellings, narrow courts, and so forth, to replace the dwellings so removed by healthy structures in all respects fit for human habitation, and to re-arrange the streets on a plan admitting of plenty of air and light."

The first step is for the medical officer of health to make a representation to the authorities, setting forth the condition of the area. He may do this of his own accord; or he may be compelled to make an inspection and give a report on the strength of a complaint lodged by two Justices, or by twelve or more ratepayers. To render this complaint valid, it is not necessary that the houses in the area complained of should be unfit for human habitation, though a large proportion of them generally are. It is enough that "the narrowness, closeness, and bad arrangement, or the bad condition of the streets and houses within such area, or the want of light, air, ventilation, or proper conveniences, or any other sanitary defects, or one or more of such causes, are dangerous or injurious to the health of the inhabitants either of the building in the said area or of neighbouring buildings." It is essential, however, to show that the sanitary defects cannot be effectually remedied otherwise than by the rearrangement and reconstruction of the streets and houses within the area.

With regard to individual houses which the sanitary authorities condemn, a magistrate's order to close the house may be obtained. When this has been granted the tenant has to leave the house, and the landlord is expected to perform such alterations and improvements, where such are possible, as will justify the magistrate in giving another order permitting the house to be occupied. If the owner does nothing or seems unduly slack in fulfilling his duty the local authority can order the demolition of the house, though any reasonable protest from the owner or explanation of his delay will be listened to and considered. It is obvious that this is a law whose efficiency depends much on the spirit in which it is administered. It is fortunate that, at least in the larger centres, public opinion is so strong in favour of erecting healthy dwellings that there is little chance of a landlord being allowed to delay improvements indefinitely, or to re-open the house without rendering it healthy and decent. In small towns and villages it is to be feared that the law is not so strictly administered; but everywhere, under the impetus given by this Act, old houses are being pulled down and better ones erected in their place; and the regard paid to sanitation, although keener in some places than others, is everywhere developing.

With regard to the requirements of a dwelling fit for human habitation, the Public Health (London) Act of 1891 may be taken as defining these, although, as is inevitable, in buildings already erected when the Act was passed, defects are tolerated which must not be repeated in new houses. For example, an Act passed in 1875 stopped the growth of cellar-dwellings; but where these were already in existence they are tolerated under certain conditions. They are permitted, where their walls are seven feet high, with at least three feet of their height above the surface of the street or adjoining ground. There must also be outside and adjoining the cellar, and extending along the entire front, and upward from six inches below the level of the floor to the surface of the street or ground, an open area of at least two and a-half feet wide. There must also be drains, closets or privies, ash-pits, and fire-places, and a window opening directly on to the air, nine feet square in size, and readily opened. If cellars, which do not come up to these moderate requirements, are let as dwellings, the owner is liable to penalty of £1 a day during their occupancy.

For new houses the following are the general regulations laid down by the London County Council, which may be taken as typical of the best-intentioned providers of working-class houses in England: Staircases must be four feet wide; dwellings must not be more than four storeys high; ceilings must be nine feet high. Blocks of buildings should not exceed forty feet in height. Parallel blocks should be separated from each other by an open space, clear from the ground, of at least forty feet in width. New streets must be forty feet in width. With regard to drainage, the County Council stipulates that one side of the water-closet, at least, must abut on a space of not less than 100 square feet. No closet may be approached directly from a living-room or a store-room for food. A window of not less than two square feet shall open directly to the external air. Bath and washhouses are to be provided for every block of dwellings. This is best done in a separate block of buildings or on the basement floor. Water-closets, also, in houses built for the lowest classes of the population, are best built outside the dwelling proper, although, if possible, there should be a closet for each family. Closets, water-supply, and sinks may be conveniently provided on each floor of the staircase, with no communication with the living-rooms of any house.

Living-rooms in one-room tenements are to be not less than 12 ft. by 12 ft. In two and three room tenements this room is supplemented by apartments measuring 12 ft. by 8 ft.; but some discretion is allowed to the architect in varying the sizes of the rooms so long as the total superficies of the house corresponds to these requirements.

* "Lectures on Sanitary Law." By A. Wynter Blyth, M.R.C.S.Eng., L.S.A.Lond., Barrister-at-Law of Lincoln's Inn, Professor of Hygiene, College of State Medicine.

